# Embedding Ba Monolayers and Bilayers in Boron Carbide Nanowires

**DOI:** 10.1038/srep16960

**Published:** 2015-11-26

**Authors:** Zhiyang Yu, Jian Luo, Baiou Shi, Jiong Zhao, Martin P. Harmer, Jing Zhu

**Affiliations:** 1 Beijing National Center for Electron Microscopy, School of Materials Science and Engineering, The State Key Laboratory of New Ceramics and Fine Processing, Laboratory of Advanced Materials (MOE), Tsinghua University, Beijing 100084, China; 2 Center for Advanced Materials and Nanotechnology, Department of Materials Science and Engineering, Lehigh University, Bethlehem, Pennsylvania 18015, United States; 3 Department of NanoEngineering, Program of Materials Science and Engineering, University of California, San Diego, California 92093, United States; 4 Department of Mechanical Engineering & Mechanics, Lehigh University, Bethlehem, Pennsylvania 18015, United States

## Abstract

Aberration corrected high angle annular dark field scanning transmission electron microscopy (HAADF-STEM) was employed to study the distribution of barium atoms on the surfaces and in the interiors of boron carbide based nanowires. Barium based dopants, which were used to control the crystal growth, adsorbed to the surfaces of the boron-rich crystals in the form of nanometer-thick surficial films (a type of surface complexion). During the crystal growth, these dopant-based surface complexions became embedded inside the single crystalline segments of fivefold boron-rich nanowires collectively, where they were converted to more ordered monolayer and bilayer modified complexions. Another form of bilayer complexion stabilized at stacking faults has also been identified. Numerous previous works suggested that dopants/impurities tended to segregate at the stacking faults or twinned boundaries. In contrast, our study revealed the previously-unrecognized possibility of incorporating dopants and impurities inside an otherwise perfect crystal without the association to any twin boundary or stacking fault. Moreover, we revealed the amount of barium dopants incorporated was non-equilibrium and far beyond the bulk solubility, which might lead to unique properties.

The crystal shape and growth rate of nanomaterials can often be controlled by adding a trace amount of dopants that are adsorbed on the growing surfaces to change the surface energies and kinetics. For example, prior studies showed that the formation of an impurity-based surficial “amorphous” films^1^ (SAFs, as a type of surface complexions, where the term “complexion” refers to the thermodynamic equilibrium state of an interface; noting that this type of complexion is neither completely crystalline nor fully amorphous despite of being named as SAFs^1^) - can change faceted particles into nanospheres^2^ or stabilize anisotropic morphology^3^. Specifically, we have recently demonstrated that adding barium oxide as an additive along with the iron-rich catalyst can help to control the growth of B-C-O nanowires^4^ and nanoplatelets^5^,^6^,^7^ to achieve unique morphologies and high yields; here, we further demonstrated that the controlled anisotropic growth is related to the formation of barium-enriched surface complexions.

In a broader context, this general method of tailoring the growth and morphology of nanomaterials via forming special interfacial adsorption structures (*a.k.a.* a class of 2D surface phase-like states that are called as “complexions” based on the argument that they are not Gibbs phases rigorously; see references^8^,^9^ for the rigorous definition) is an example of complexion engineering, which refers to the use of desirable interfacial complexions to control the morphological and microstructural development and materials properties^1^,^8^,^9^.

On the other hand, it is now known that the adsorbed dopants at the surfaces can be trapped into the bulk in the form of isolated atoms during the crystal growth. This phenomenon, termed “solute trapping,” was first reported in splat-quenched alloys in 1969^10^ and systematically studied in the 1980s, facilitated by the invention of pulsed-laser melting techniques^11^,^12^,^13^,^14^. High concentrations of trapped solute atoms inside the crystal matrix were confirmed in alloys^15^ and ion implanted silicon^12^,^13^ by Rutherford backscattering spectrometry (RBS) with a resolution of several nanometers. The direct visualization of isolated solute atoms in nanowires at the atomic resolution has been achieved by several groups in the last few years. Quantitative analysis of catalyst incorporation revealed that a colossal amount of catalyst atoms were injected into nanowires^16^,^17^, as confirmed by atomic probe tomography (APT). Li *et al.* discovered by HAADF focal series imaging that a high concentration of isolated Au atoms above the solubility could be buried in silicon nanowires^18^. More interestingly, aided by aberration corrected microscopy, a few studies independently provided direct evidence of an additional form of impurity atoms trapped in the bulk – 2D impurity layers could be collectively incorporated at twin boundaries^17^ and stacking faults^19^,^20^. The stabilization of impurity layers in those planar defects is suggested as a common impurity segregation phenomenon in the nanowires^17^.

Here, using aberration-corrected, high angle annular dark field scanning transmission electron microscopy (HAADF -STEM), we demonstrated that barium-enriched surface adsorbates (a surface complexion) that were used to control the shape and growth of B-C-O nanowires^4^,^21^ can also be collectively trapped inside the crystals and converted to 2D layers of barium atoms. Specifically, three types of 2D embedded layers with different atomic structures were identified, two of which are newly-discovered forms of 2D impurity layers that were trapped inside an otherwise perfect crystal without the association with any twin boundary or stacking fault. These two types of embedded layers cannot be explained by Chen *et al.*’s model^17^, where it was proposed that impurities were initially incorporated uniformly in the bulk and subsequently segregated to the planar defects; instead, they should be regarded as a result of the non-equilibrium trapping and conversion of impurity based surface complexions.

Using quantitative electron microscopy, we found an extremely high level of dopant incorporation above the bulk solubility within nanowires. Moreover, we showed that the degree and extent of dopant layers could be controlled by manipulating the processing temperatures. This study directly demonstrated that 2D dopant/impurity layers could be embedded in crystals with a high concentration and in a controlled fashion, revealing new possibilities for creating ordered 2D impurity/doping internal layers.

## Results

### The structure of surface complexions

**Figure 1:**
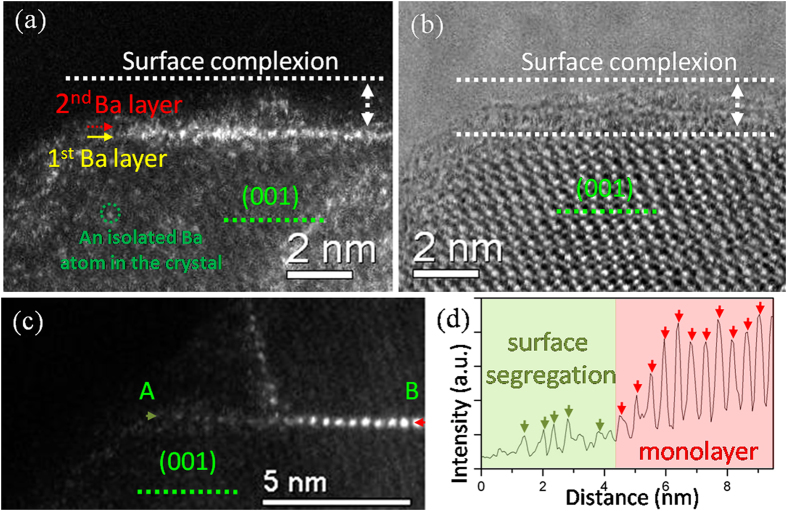
(**a,b**) A pair of HAADF and bright filed (BF) images showing the structure of surface complexions on the {001} surfaces. The complexion thickness ranges from 0.9 to 1.3 nm. (**c**) Direct observation of the transition from a partially ordered surface segregation to an ordered Ba monolayer trapped in a single-crystal nanowire. (**d**) A HAADF-STEM line profile was taken along this transitional region from spot A to spot B in panel (**c**), showing the changes in ordering. The arrows indicate the positions of Ba columns.

The addition of BaO is critical in the formation of boron-rich fivefold twinned nanowires, which are mainly enclosed by {100} ((100), (010) and (001) planes are identical for boron carbide crystal with rhombohedra symmetry) planes, as shown in Supplementary Fig. S1. Without BaO in the starting precursor, the resulting boron-rich crystal was irregular in crystal shape, suggesting that the surface energies were nearly isotropic. Hence, BaO, as a growth habit modifier, induced anisotropic growth of boron carbide crystals. The exposed {100} surfaces after Ba doping were capped by Ba-enriched, nanometer-thick surface complexions, as revealed by aberration corrected STEM (Fig. 1(a,b)). Since barium is much heavier than the lightweight matrix, Z contrast (HAADF) imaging provided valuable information on the complexion structure and its chemistry. A partially-ordered layer of barium was directly attached to the {001} surface. The first layer of Ba atoms on {001} terrace were confined in the direction vertical to {001} planes but have more freedom to relax laterally. The HAADF contrast in the complexion decreased significantly approaching the vacuum, with sparsely distributed bright spots in a dark background. This is an indication that the Ba atoms in the second layer have a much lower number density and exhibit less structural ordering.

The fabricated nanowires were generally covered by Ba-enriched nanolayer complexions on all the surfaces, as evident in high resolution electron microscopy (Supplementary Fig. S2). Electron energy loss spectra (EELS) recorded on the glassy pocket and the surface complexions with edge-on conditions showed evident Ba signals, confirming the presence of Ba (Supplementary Fig. S3). In the bulk regions, shallow Ba peaks were also detected due to the presence of 1 ~ 2 nm nanolayer complexions on the surfaces. Interestingly, iron bromide, which was used as metallic catalysts for high yield syntheses, was below the detection limit of the EELS and could be regarded as insoluble in the nanolayer complexions (Supplementary Fig. S3). Combining all these facts together, we verified that the {100} surfaces of boron-rich crystal was covered by a partially-ordered barium layer, followed with a nanometer thick B-C-O film that accommodated some dilute and less-ordered Ba atoms (amorphous-like in HRTEM).

### High Ba concentrations in the incorporated monolayers

Surprisingly, those partially-order barium atoms on {001} terraces were randomly incorporated inside the crystalline variants of fivefold twined nanowires (Fig. 1(c,d) and Supplementary Fig. S4) as partial segments but not full layers along the thickness direction. The incorporated barium atoms appeared to be more ordered than the barium atoms on the adjacent {001} terraces, implying a collective re-arrangement and ordering of the impurity atoms within the crystal. The significant contrast difference between the incorporated zone and the surface complexions (Fig. 1(d)) implied that the barium concentration in the incorporated monolayer complexions exceeded that in the surface complexions.

**Figure 2:**
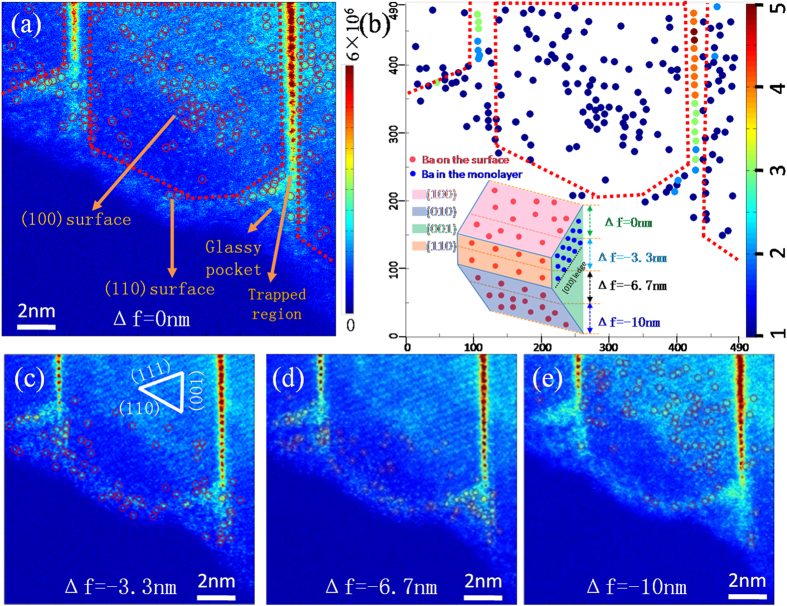
Frames of through-focal HAADF images on an ultrathin crystal segment showing isolated Ba atoms adsorbed on the top surface (**a,c**) and the bottom surface (**d,e**). By gradually underfocusing the probe, single barium atoms mainly on the (100) top surface (panel (**a,c**)) and on the (010) bottom surface (panel (**d,e**)) were resolved, with a schematic illustration of the position of the focal plane in the inset of panel (**b**). (**b**) The numbers of Ba atoms contained in the dotted regions of **(a)** are extracted and plotted as a function of their positions.

Additionally, quantitative electron microscopy has been employed to study the local concentration of barium atoms within the nanowires and on the surfaces of the nanowires (Fig. 2). The detailed quantification procedure is narrated in the supplementary information (Section 2). In brief, three types of Ba atoms (those in the trapped monolayer and surface complexions as well as isolated Ba atoms inside the crystal) were detected and their corresponding barium concentrations could be estimated as follows. In brief, the atoms inside the red dotted regions in Fig. 2(a) could be either ascribed to the impurities on surfaces or the impurities within the bulk. We cannot distinguish between Ba atoms on the surfaces and in the bulk owing to the poor depth resolution of HAADF-STEM (several nanometers). In spite of this, on one hand, we could assume that all the 269 atoms were on surfaces (Fig. 2(a–e) and Supplementary Table S1, 142 + 20 + 23 + 84 = 269), placing an upper bound limit of Ba coverage of 0.84 aotm/nm^2^ within the (100) and (010) nanolayer surface complexions. On the other hand, by assuming all the 269 atoms were trapped in the crystals, an upper bound limit of the solubility of Ba in the bulk is established as (20 + 23)atoms/80 nm^3^*0.446 nm = 0.24 atom/nm^2^, where the number of 0.446 is the interplanar spacing of (001) surface. As a matter of fact, the numbers of Ba on the surface were believed to be close to the reality and therefore the actual solubility of Ba atoms in the bulk is greatly overestimated. We arrived at this judgment for the reason that, as shown in Fig. 1(a), from the side view, the isolated atoms trapped within the crystal showed much lower contrast than the ones in surface complexions, suggesting that much fewer numbers of Ba were trapped in the crystal compared with those remaining on the surface. The atoms contained in the trapped monolayer could be calculated by normalizing their Gaussian peaks with the average of the Gussian peaks from all single Ba atoms, considering that the trapped columns contained a small number of Ba atoms and thus the HAADF intensities are approximately linear to their atomic densities. The number of Ba atoms trapped in the monolayer is 85 (Supplementary Table S1), giving rise to a Ba concentration of 1.75atom/nm^2^ in the monolayer. Hence, quantitatively, the Ba monolayer incorporated more solute atoms than those contained with the surface complexions and in the bulk.

### Structural details of monolayers and bilayers

**Figure 3:**
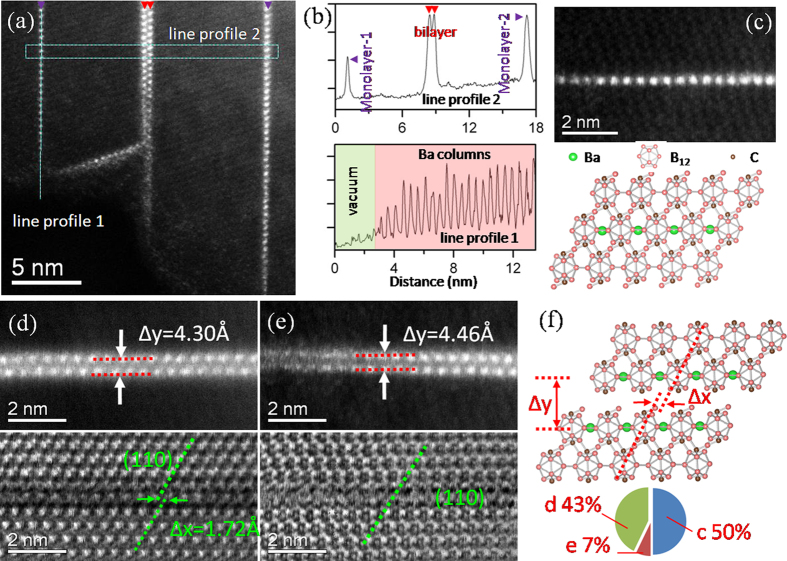
(**a**) A low magnified HAADF image showing the coexistence of monolayers and bilayers in a crystalline segment. (**b**) Corresponding line profiles along a buried monolayer and across trapped planar dopants. (**c**) A typical micrograph showing a Ba-enriched monolayer trapped in a single crystal. Pairs of typical HAADF and BF images showing cases of a Ba bilayer trapped in a stacking fault (**d**) and a bilayer (**e**) buried in a single crystalline segment. **(f)** A schematic of a Ba bilayer in a stacking fault. When Δx is close to zero and Δy is identical with the (001) interplanar spacing of boron carbide, the bilayer was determined as a planar impurity buried in a single crystal, otherwise they were considered to be associated with stacking faults. The inset of (**f**) shows the frequency of the three different solute trapping behaviors in this study (c for monolayers in single crystals, d for bilayers in stacking faults and e for bilayers in single crystals).

More generally, there were two basic forms of Ba layers that were highly ordered in the bulk: bilayers and monolayers (Fig. 3(a)). A line profile was taken across those two types of layers (Fig. 3(b), upper panel). The impurity densities in monolayers and bilayers were close as evident by the comparable HAADF intensities. Inspection of the line profile along a monolayer revealed additional information (Fig. 3(b), lower panel): the atomic column intensities of impurity did not increase monotonically when entering into the bulk but they were almost a constant with local intensity variations. This is an indication that nearly identical amounts of impurity atoms in the depth direction were collectively incorporated in the monolayer, although it sounds somewhat counterintuitively. A schematic of the cross-section structure of the trapped Ba atoms in the monolayer deduced from focal series imaging was shown in Supplementary Fig. S5. We examined 29 sets of white lines (corresponding to incorporated Ba layers, Supplementary Fig. S6) in a 1100 °C nanowire and found that, 50% of the planar impurities were monolayers that were buried in single crystals (Fig. 3(c)); ~43% were bilayers which were associated with stacking faults (Fig. 3(d,f)); the remainder (~7%, Fig. 3(e)) was trapped as bilayers in single crystals. Our statistical analysis on the structure of impurity layers revealed two unrecognized forms of dopant atoms in the crystal: monolayers and bilayers incorporated in the single crystals.

The incorporation of Ba monolayers observed in this study clearly does not follow Chen *at al.’s* defect-segregation model^17^ because they were not associated with crystal defects. In the current case, all the monolayers were parallel to the interface between the iron-rich catalyst and the nanowire (see Supplementary Fig. S4). It was suggested that the formation of monolayers could be better explained by the notion that the lateral motion of ledges is so fast that a whole Ba layer originally adsorbed on (001) terraces is entirely buried. After the incorporation, the crystalline environment will likely induce a collective redistribution of impurities, leading to ordered Ba columns. In some less-common cases, a fast moving ledge with a height that is twice the {001} lattice spacing may propagate swiftly, leading to the incorporating of not only the 1^st^ layer of dense Ba atoms but also the 2^nd^ dilute Ba layer (see the definition of Ba layer in Fig. 1(a)) and finally giving rise to the Ba based bilayers buried in the single crystal. Occasionally, some barium atoms in the bilayers did not have time to re-arrange into an ordered structure and stayed in a more disordered pattern (Fig. 3(e)). The discovery of monolayer and bilayers reveals additional forms of non-equilibrium 2D impurity based defects and suggests new possible dynamic processes for the interactions between step motions and solute atoms on the surface.

Stacking faults were frequently observed in boron-rich crystals and they were considered to arise from the instability in the iron boride catalysts, which assisted the continual nucleation of (001) ledges^7^. In some cases, we also observed trapped bilayers stabilized at stacking faults, similar to the situation of impurity segregation at grain boundaries^17^ (see Fig. 3(d,f)). Here, We consider two possible mechanisms for the formation of bilayers at the stacking faults: first is that they formed according to Chen *et al.*’s defect-segregation model where the enrichment of Ba bilayers is ascribed to the preferential segregation at the defects; second is that the surface Ba layers (complexions) were first trapped in the stacking faults and then converted to ordered impurity arrays. A more conclusive mechanistic explanation is unwarranted at this time.

### Implication of layer trapping

**Figure 4:**
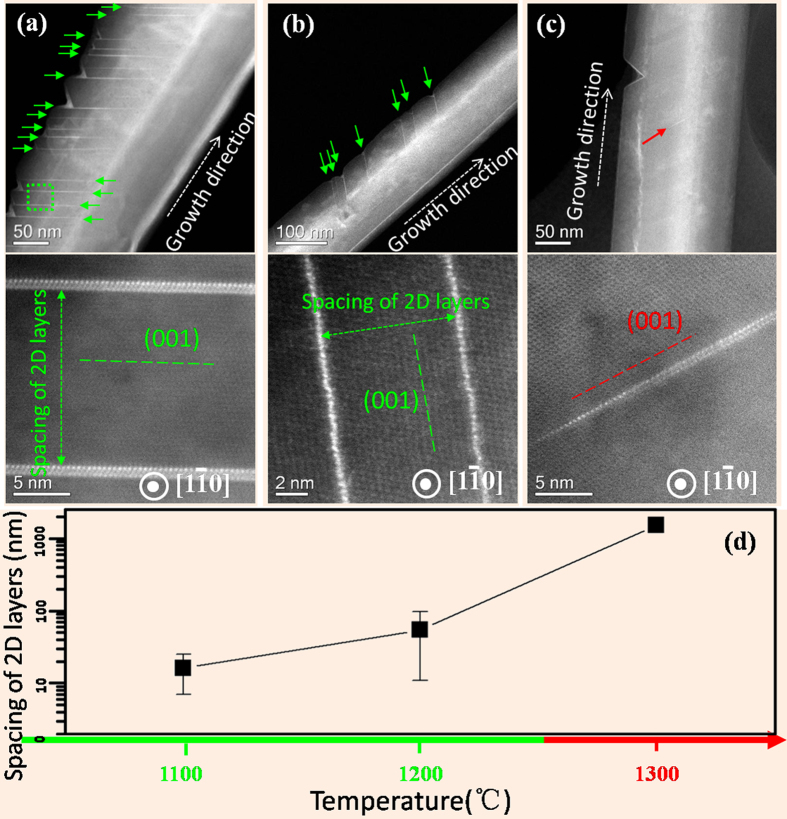
Low magnification HAADF images of nanowires (upper panels) and corresponding expanded view (lower panels) of trapped 2D barium layers buried within fivefold twined nanowires synthesized at the nominal temperatures of 1100 °C**(a)**, 1200 °C **(b)** and 1300 °C **(c)**, respectively. All the nanowires were aligned to the same [1–10] orientation. (**d**) Dependence of the average spacing of 2D impurity layers on the processing temperatures. At 1300 °C, the observation of 2D layer trapped within the crystal was rare; in this specific case, we only found two trapped 2D layers (separated by a few microns) in one of the five nanowires examined.

The formation of the incorporated three types of dopant layers could be controlled (at least to some extent) by manipulating the processing temperatures (1100–1300 °C). As shown in Fig. 4, high densities of bright lines indicate that Ba layers were commonly present in the crystalline matrix at lower temperatures (1100–1200 °C). The existence of 2D layers became extremely rare at higher temperatures (1300 °C). As illustrated in Fig. 4(d), the occurrence of 2D Ba layers decreased greatly at higher processing temperatures. This behavior could be rationalized since Ba solute atoms were easier to escape the trapped zone during the fast ledge propagation at higher temperatures.

## Discussion

The current study represents one case of trapping dopant-based surface complexions by the rapid advancement of ledges. As discussed above, several prior studies already showed impurity based 2D monolayers trapped in stacking faults and twin boundaries but did not recognize the role of surface complexions during the monolayer trapping. Anomalous Eu layer doping^19^ was confirmed by high resolution HAADF-STEM and TEM-EDS in AlN ceramic powders. The growth of the powders was dominated by a step-flow growth mechanism as indicated by the high density of steps on the surfaces, which were coated by nanolayer surface complexions. It was also noticed that the co-doping of silicon was necessary for the formation of Eu layers in the bulk^19^. Considering the doping of Si in ceramics often promotes the formation nanolayer interfacial complexions, it is possible that the solubility of Eu in such surficial films can be significantly increased; subsequently, by rapid ledge propagation, a whole layer of Eu was trapped into the AlN matrix, leading to a blue luminescence under UV and electron excitations. Ce layer doping has been reported by Xu *et al.* in α-Sialon with the presence of Ce-enriched amorphous intergranular films at grain boundaries^20^. The general existence of nanolayer surface complexions in these specimens^19^,^20^ suggests that a similar trapping-surface-complexion mechanism could operate in incorporating monolayers at stacking faults in those two systems, but a further investigation has to be conducted to verify this hypothesis. If the hypothesis is confirmed, those studies^19^,^20^ also suggest that converting surface complexions into embedded ordered 2D impurity layers is an effective method for impurity doping in other systems, with beneficial effects on the luminescence properties.

In summary, HAADF-STEM revealed the trapping of barium atoms in boron carbide based crystals, where a significant amount of Ba atoms were present in the form of ordered 2D monolayers or bilayers. We believe that these monolayers/bilayers were trapped surface adsorbates (in the form of Ba-enriched SAFs) that ordered during the trapping processing. Those barium-enriched 2D layers have significantly higher effective local concentrations than that of the bulk, which may have a significant influence on many properties.

## Methods

A chemical vapor deposition (CVD) method has been developed for the mass-preparation of B-C-O nanowires with fivefold twinned cross-section^6^,^22^ and platelets^4^ with parallel twinned cross-section using BaO, B and Fe_3_O_4_ as precursors. The processing temperature was set to 1100–1300 °C to fabricate nanowires as the main product. It is believed that the nanowires grew during the cooling stage (since the growth of nanomaterials is not affected by duration of the isothermal annealing generally); thus, the processing temperature is only nominal. The chemical compositions of the nanowires were close to B_4_C with minor amount of oxygen. Here, we refer to them as boron carbide based nanowires for the sake of simplicity. Nanowires were harvested and transferred onto carbon holey films for TEM characterization. Sub-angstrom HAADF imaging was conducted on a JEOL 2200FS microscope installed with a probe aberration corrector.

## Additional Information

**How to cite this article**: Yu, Z. *et al.* Embedding Ba-monolayers and Bilayers in Boron Carbide Nanowires. *Sci. Rep.* **5**, 16960; doi: 10.1038/srep16960 (2015).

## Supplementary Material

Supplementary Information
