# Supplementary material for: Embedding Ba Monolayers and Bilayers in Boron Carbide Nanowires
Source: Sci Rep. 2015 Nov 26;5:16960. doi: 10.1038/srep16960 (PMC4660277; doi:10.1038/srep16960)
Supplement: Supplementary Information [file srep16960-s1.doc]

# Supplementary Information

**Embedding Ba Monolayers and Bilayers in Boron Carbide Nanowires**

Zhiyang Yu^1,2^, Jian Luo^3^, Baiou Shi^4^, Jiong Zhao^1^, Martin P. Harmer^*,2^ & Jing Zhu^*,1^

^1^ Beijing National Center for Electron Microscopy, School of Materials Science and Engineering, The State Key Laboratory of New Ceramics and Fine Processing, Laboratory of Advanced Materials (MOE), Tsinghua University, Beijing 100084, China

^2^ Center for Advanced Materials and Nanotechnology, Department of Materials Science and Engineering, Lehigh University, Bethlehem, Pennsylvania 18015, United States

^3^ Department of NanoEngineering, Program of Materials Science and Engineering, University of California, San Diego, California 92093, United States

^4^ Department of Mechanical Engineering & Mechanics, Lehigh University, Bethlehem, Pennsylvania 18015, United States

E-mail: [mph2@lehigh.edu](mailto:mph2@lehigh.edu), [jzhu@mail.tsinghua.edu.cn](mailto:jzhu@mail.tsinghua.edu.cn). Telephone: +86 10 62794026. Fax: +86 10 62772507

## Section 1: Figures


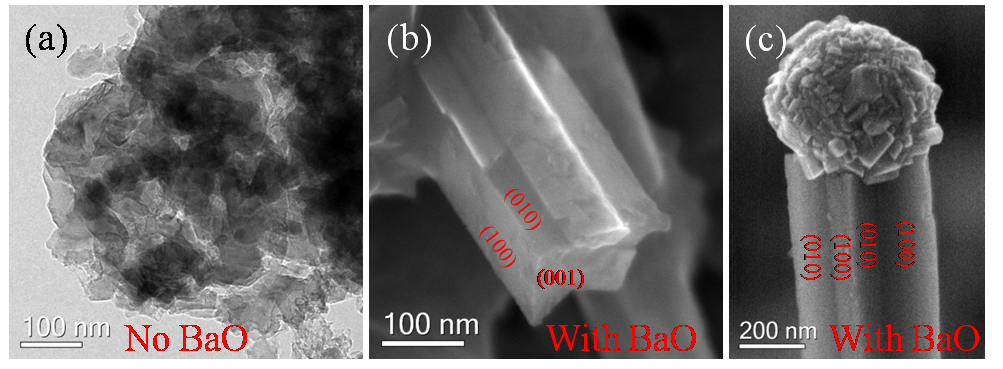


**Figure S1** **(a)** Bright field (BF) TEM images of boron-rich irregular flakes fabricated without using BaO as an additive. The surfaces of flakes were rough and curved. **(b, c)** The addition of BaO significantly promoted the anisotropic growth of fivefold twinned nanowires, which were mainly terminated by {100} facets.


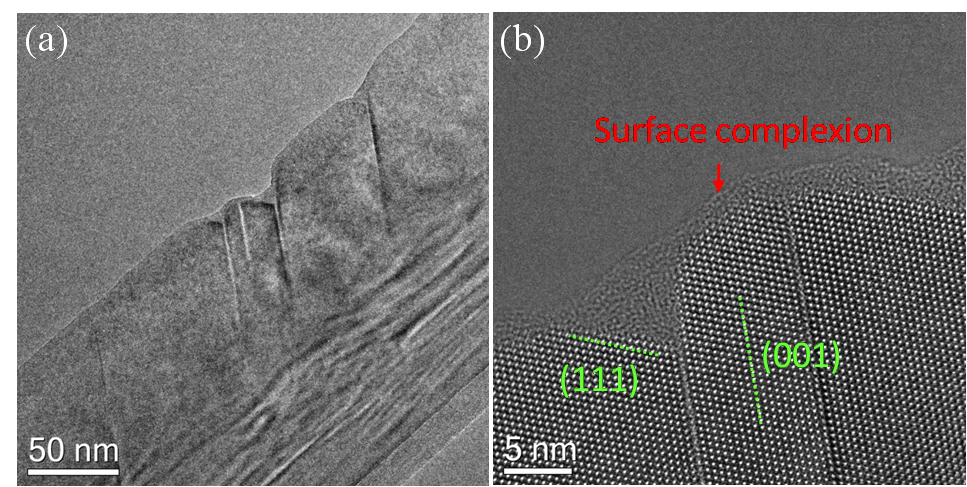


**Figure S2** **(a)** Low magnification TEM image of a boron carbide based nanowire. **(b)** An enlarged high resolution TEM image showing that the surface was covered by 1-2 nm thick nanoalyer surface complexions. Due to the presence of multiple facets and glassy pockets, this disordered film did not have a constant thickness (in contrast to the constant-thickness complexions formed on flat surfaces).


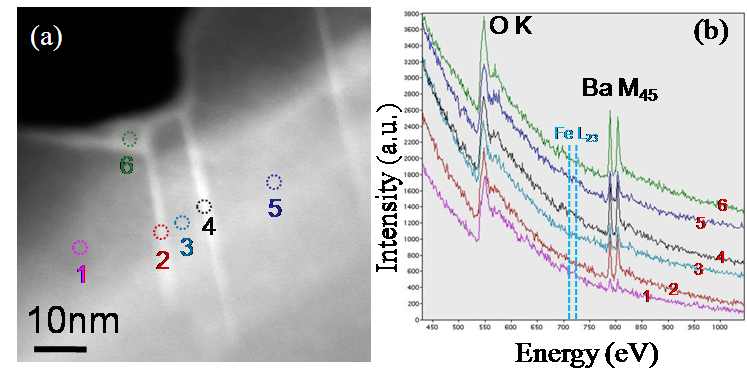


**Figure S3** **(a)** A low magnification HAADF image of a naonwire with Ba monolayers/bilayers orientated to an edge-on condition. **(b)** EELS spectra were recorded at the bulk sample (spots 1, 3 and 5), at the trapped layers (spots 2 and 4) and at the glassy pockets (spot 6). Sharp Ba M_45_ peaks were evident on the trapped layers and the glassy pockets (~5~20 nm in thickness direction), suggesting that Ba is a necessary constitutional element of the glassy phases and the trapped complexions. This observation is consistent with the high HAADF contrast at the trapped regions. Shallow Ba peaks were discernable from the points on the bulk sample and the signals actually arose from the thin surface amorphous film (1-2 nm in thickness) on the top and bottom surface. In contrast, there is no evidence for the existence of iron in any region. Hence, it is confirmed by EELS that the nanowire was capped with Ba-enriched nanolayer surface complexions.


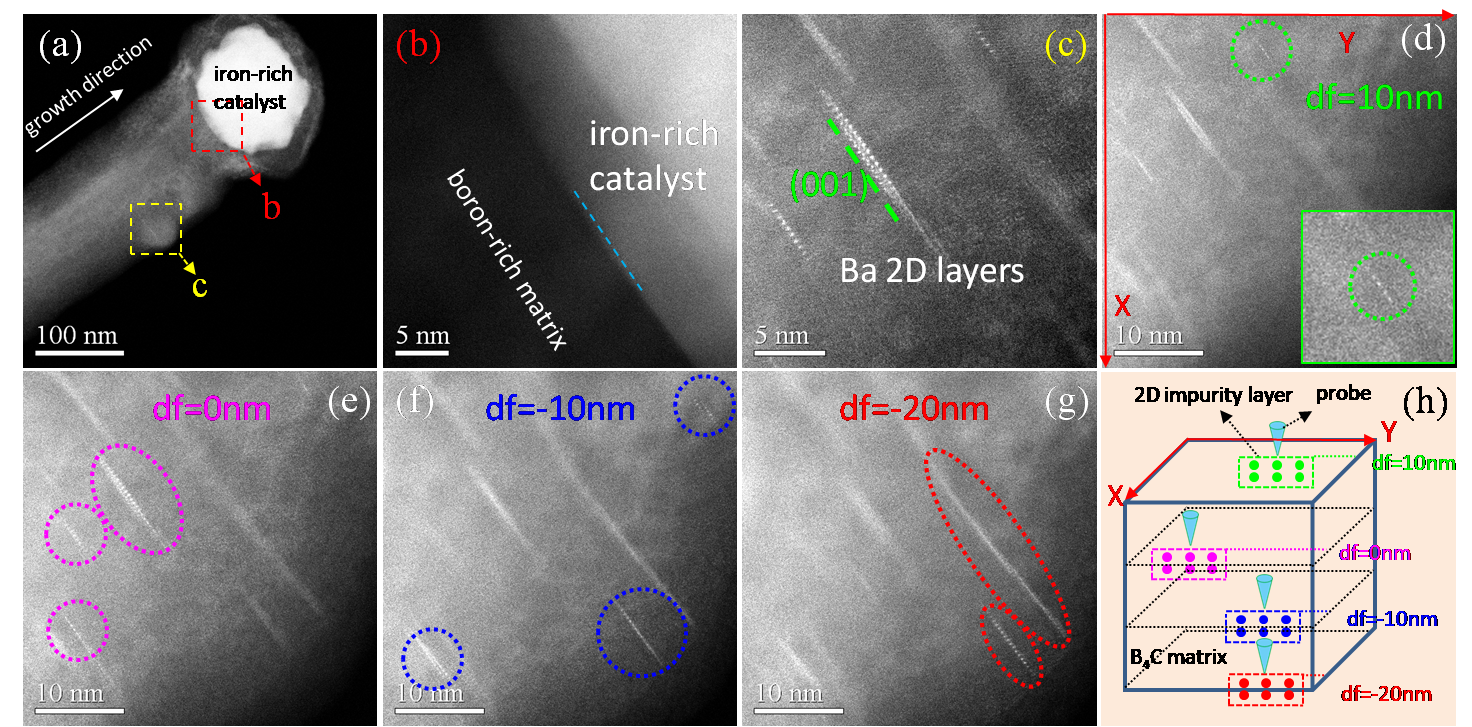


**Figure S4** **(a)** Low magnification HAADF image of a typical nanowire capped with a catalyst which has an iron-rich core and a barium-enriched B-C-O crystalline shell. The shell of the catalyst crystallized to a boron carbide matrix in the final stage of growth and it is difficult to see the barium-enriched surface complexions sitting on the growth front of the boron carbide nanowire. Nevertheless, the interface between the nanowire and the catalyst was persevered (**(b)**). A set of white lines were in parallel with this interface, indicating that the trapped impurity layer originated from the surface complexions. A typical high magnification HAADF image showing trapped 2D layers within boron-rich matrix is given in panel **(c)**. **(d-g)** Focal series images of 2D impurity layers inside the boron-rich crystal. The 2D layers that were in focus at each frame were marked out by circles. For example, a minor amount of Ba atoms were trapped in the 2D layers as indicated by the green circle in **(d)**. An enlarged view of the circled region was shown to highlight the 2D Ba layer in the inset of **(d)**. Those impurity layers came into focus at different defocus values. For example, the 2D layers indicated by pink circles were resolved at a defocus value of 0 nm while they were immediately out of focus at defocus values of 10 nm and -10 nm. The barium 2D layers were trapped as partial segments but not full layers along the thickness direction, and their depths varied greatly. **(h)** To help a better understanding of the three-dimensional distribution of impurity layers, a schematic illustration was plotted.


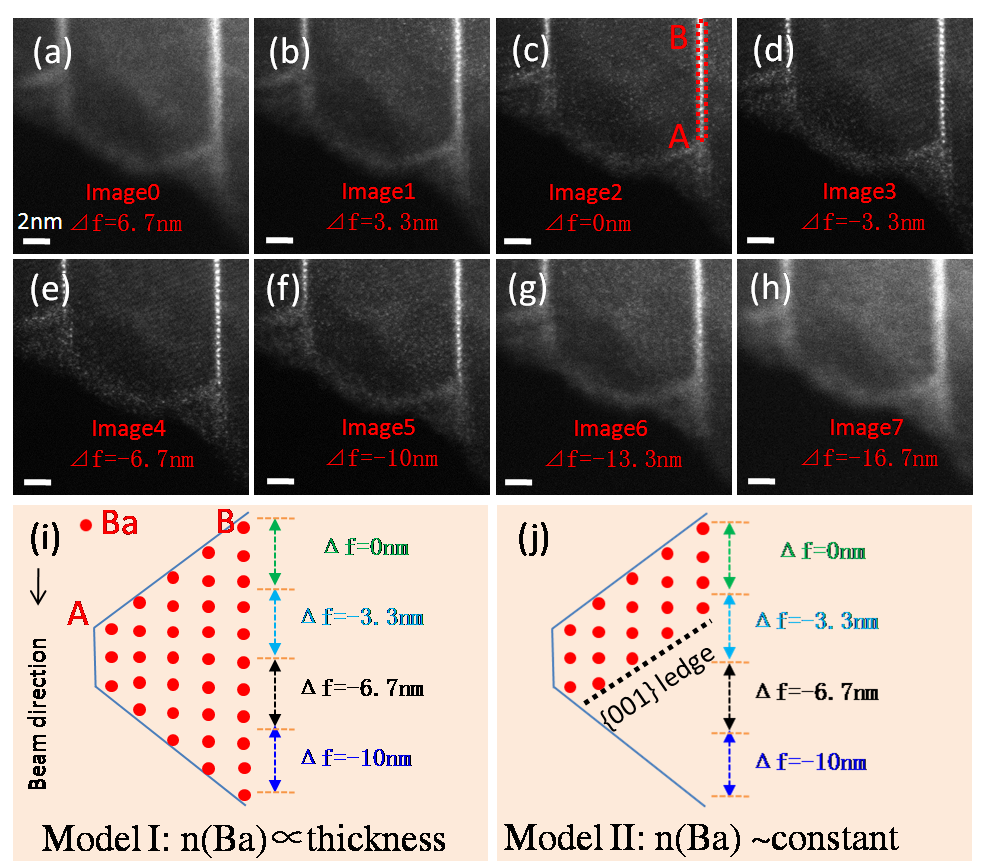


**Figure S5** **(a-h)** Original through-focal HAADF images of Figure 2. Here we show the full frames. The image drifts in these images have been corrected. No further image processing was applied on these images. **(i-j)** The schematics of two possible models with Ba atoms trapped in the crystal. Here we present them in the cross-section views. In **(i)** the numbers of Ba atoms in the beam direction increase steadily as the thickness of the nanowires increases. In **(j)** the numbers of Ba atoms in the beam direction are almost constant with a minor local variation. The first model contains an increased numbers of Ba atoms at thicker crystals while in the second model; the amount of Ba atoms is almost constant irrespective of the sample thickness. As shown in **(a-h)**, as we gradually moved the focal plane downward, the top of the monolayer (region B in **(c)**) came into focus at first (**(b)**), and at a focal value of 0 to -6.7 nm (**(c)-(e)**), the monolayer was clearly resolved including the top and the bottom. Later, in frame **(f)**, the bottom part (region A in **(c)**) was in focus. Finally the monolayer was out of focus when we underfocused the object lens by another 3.3 and 6.6 nm (**(g-h)**). The focal series images directly point to model II but not model I. If it has a similar atomic arrangement as model I, the top region (B) would come into focus first, then the bottom region (A) would be at the focal plane, and finally the region A would come out of focus before the region B is dim. This was not observed in the focal series images and we believe the real atomic packing in the trapped region is more close to model II.


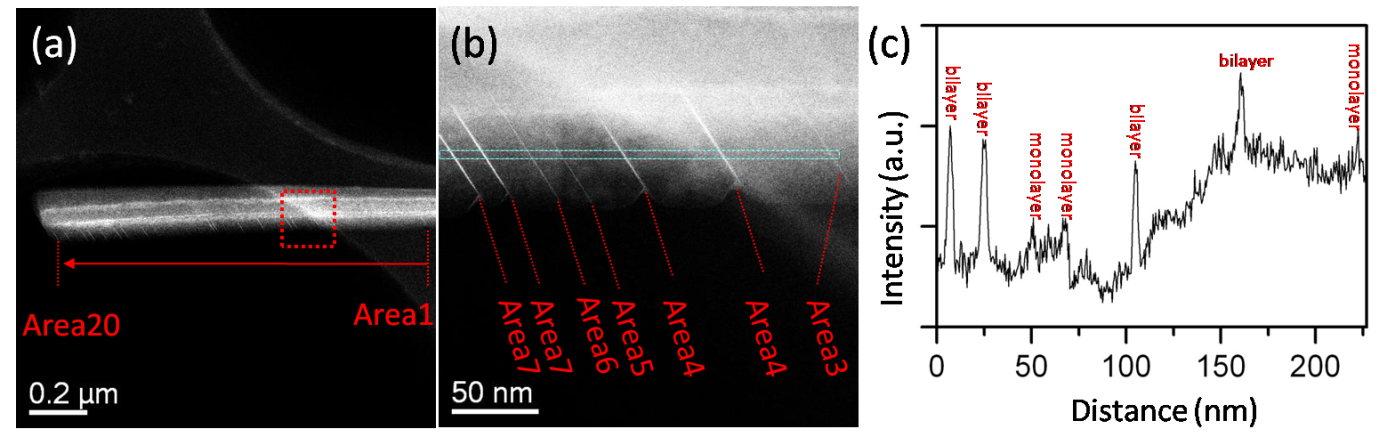


**Figure S6** **(a)** Overview of the 1100 ℃ nanowire studied in Figure 1-3. The regions which contained bright lines were labeled as area 1-area 20. **(b)** An expanded view of several typical regions. A line profile across the barium-rich bright lines was shown in **(c)**. The intensities of bilayer and monolayer were comparable, indicating that they have about the same amounts of Ba atoms trapped.

## Section 2 Quantification of Ba concentration in the surface complexions and in monolayers

### 2.1 The effect of sample thickness on the detection of isolated Ba atoms on the surface

It has been well established that the intensity of atom columns (*I*) approximately reflects their mean square atom number (*Z*) and here we assume the intensity is proportional to *Z*^1.7[1]^. A simple calculation indicates that the intensity of a single Ba atom will be equivalent to that obtained from a boron carbide nanowire with a thickness of 16.8nm, which corresponds to 60 boron atoms in the beam direction. In reality, since boron and the nearest carbon atoms has a short bond length (along [1-10] projection, <1.4Å), these two columns together contribute to a bright spot in the acquired experimental HAADF image, making the critical sample thickness for imaging single Ba atoms below 10nm.The wedges of boron-rich nanowires were quite thin (with a thickness less than 10nm in most cases), which facilitated the detection of single barium atoms on their surfaces. Once the sample thickness surpasses this limit, the signal of isolate Ba atom is comparable with that from the matrix, especially when the probe is focus on the lattice. As shown in the thicker part of the nanowire in Fig. 2(c,d), the detection of single Ba atoms starts to become difficult since the lattice itself also contributed to high HAADF intensities.

### 2.2 The effect of electron beam damage on counting the single atoms on the surface

The intensity of Cs-corrected probe was quite strong and thus isolated barium atoms, especially on the surfaces were vulnerable to beam damage and more possibly, they would suffer electron driven migration on the surfaces. We have conducted a pre-test on beam damage and beam driven surface migration on isolated Ba atoms and we found, 70% of the atoms stayed on the original sites after a continuous probe scanning for 30s. The rest 30 % of the atoms either migrated on the surfaces several nanometers off their original sites or suffered beam damage. Each frame of the focal series images was recorded within 8 seconds, and as a result, all the focal series images were captured within ~32 s. Hence, although ~30 % of the atoms would suffer beam damage or beam induced migration, the general picture on the quantification of single Ba atoms within surface complexions will not change much.

### 2.3 Gaussian fitting of HAADF images

Before we perform 2D Gaussian fitting on the bright spots associated with Ba atoms, stage drifts have been corrected for each frame of focal serial images via a script based on convolution method (Fig. 2 and Supplementary Fig. S5).

After drift correction, a ~15 by 15 image with the brightest pixel (isolated Ba atom) in the center was cropped and later it was fitted into a 2D Guassian functions by a script.

The cropped images were imported as matrices and fitted as two dimensional Gaussian functions:

*I* = *I*_gb_ + *I*_signal_ * exp(-(*x* - *x*_0_)2/w_1_^2^ - (*y* - y_0_)^2^ / w_2_^2^)

Where *I*_gb_, *I*_signal_ were the fitted background, fitted Gaussian peak; x_0_, y_0_ were the Gaussian peak centers and *w*_1_ and *w*_2_ were the Gaussian widths. A filter has been adopted to judge the goodness of fit (*w*_1_ < 7 (corresponds to a Gaussian width of 2.4 Å), *w*_2_ < 7 and *R*-square (goodness of 2D Gaussian fit) > 0.3). Some isolated Ba atoms got damaged by the electron beam during the HAADF-STEM imaging and the corresponding cropped images could not pass the filter.

The centers of isolated barium atoms were marked out by solid red circles. Interestingly, the intensity of the Gaussian peaks of isolated atoms on surface were within a small range of 0.9-1.2 × 10^6^, the average of which could be treated as a nominal Gaussian intensity of a single Ba atom. The intensities of all the barium columns in the monolayers were normalized using this number and the numbers of Ba atoms trapped were extracted in Fig. 2(b). In order to make single atoms evident in the HAADF, we intentionally saturated the super bright pixels associated with Ba monolayers in Fig. 2. The original HAADF images were given in Supplementary Fig. S5.

### 2.4 Quantification of Ba concentrations

Before we calculated the Ba concentration within the surface complexions and within the trapped regions, it is important to understand how surface complexions were coated on the surfaces of the nanowires. First, as shown in Fig. 2(a), the edge of the nanowire was terminated by a (110) surface (standing edge-on), on which the detected single atoms should be excluded in the calculation of the Ba concentration on {100} surfaces. Secondly, the solute atoms in the glassy pocket should be eliminated from the calculation. Third, there are still three types of Ba atoms to be considered: those in the trapped monolayer, surface complexions and the bulk.

Owing to the poor depth resolution of HAADF-STEM we cannot distinguish between Ba atoms on the surfaces and in the bulk. For example, the atoms inside the red dotted box in Supplementary Fig. S5(a) could be either ascribed to impurities on surfaces or within the bulk. Here, we could assume that all the 269 atoms were on surfaces (Supplementary Table S1, 142+20+23+84=269), placing an upper bound limit of Ba coverage of 0.84aotm/nm^2^ within the (100) and (010) nanolayer surface complexions. On the other hand, by assuming all the 269 atoms were trapped in the crystals, an upper bound limit of the solubility of Ba in the bulk is established as (20+23)atoms/80nm^3^*0.446nm=0.24 atom/nm^2^, where the number of 0.446 is the interplanar spacing of (001) surface. As a matter of fact, the actual solubility of Ba atoms in the bulk is negligibly small. We arrived at this judgment for the reason that, when we set the surface complexions to edge-on conditions (Fig. 1(a)), the isolated atoms trapped within the crystal have much lower contrast than the ones in surface complexions.

The atoms contained in the trapped monolayer could be calculated by normalizing their Gaussian peaks with the average of the Gussian peaks from all single Ba atoms, assuming that the trapped columns contained a small number of Ba atoms and thus the HAADF intensities are approximately linear to their atomic densities, which could be true in the current study. The number of Ba trapped is 85 in the monolayer (Supplementary Table S1), giving rise to a Ba concentration of 1.75atom/nm^2^ in the monolayer.

**Table S1** Number of single Ba atoms detected in the focal series frames of Figure 2

| **Image source** | **Number of Ba atoms in the regions outlined by red dotted regions** | **Number of Ba atoms trapped in the monolayer** |
| --- | --- | --- |
| **Fig. 2 (a)** | 142 | 85 |
| **Fig. 2 (c)** | 20 | - |
| **Fig. 2(d)** | 23 | - |
| **Fig. 2(e)** | 84 | - |

It should be noted that, we couldn’t direct image the surface complexions at the (001) growth front, owing to the fact that, most of the time, the ends of boron-rich nanowires were capped with an iron-rich catalyst. However, considering the fact that all the {001} planes are identical in a rhombohedral lattice, it is reasonable to compare the Ba distributions on the (100) and (010) surface complexions to those trapped in the bilayers/monolayers.

## References

[1] Hillyard S., &John S. Detector geometry, thermal diffuse scattering and strain effects in ADF STEM imaging. *Ultramicroscopy* **58,** 6-17 (1995).
